# Maternal LINE-1 DNA Methylation and Congenital Heart Defects in Down Syndrome

**DOI:** 10.3389/fgene.2019.00041

**Published:** 2019-02-06

**Authors:** Ivana Babić Božović, Aleksandra Stanković, Maja Živković, Jadranka Vraneković, Vesna Mahulja-Stamenković, Bojana Brajenović-Milić

**Affiliations:** ^1^Department of Medical Biology and Genetics, School of Medicine, University of Rijeka, Rijeka, Croatia; ^2^Laboratory for Radiobiology and Molecular Genetics, Vinča Institute of Nuclear Sciences, University of Belgrade, Belgrade, Serbia; ^3^Department of Gynaecology and Obstetrics, Clinical Hospital Centre Rijeka, University of Rijeka, Rijeka, Croatia

**Keywords:** DNA methylation, LINE-1, congenital heart defects, Down syndrome, maternal risk

## Abstract

**Background:** Down syndrome (DS) is one of the most common chromosomal abnormalities associated with congenital heart defects (CHD), with approximately 40 to 60% of cases showing cardiac defects. This study assessed (i) the association between maternal LINE-1 methylation and the occurrence of CHDs in children with DS and (ii) the impact of endogenous maternal factors (*MTHFR* C677T polymorphism and maternal age) and exogenous maternal factors (cigarette smoking, alcohol intake, medication use, body mass index and dietary habits such as folate intake) on maternal LINE-1 methylation and on the occurrence of CHD in children with DS.

**Patients and Methods:** The study included 90 mothers of children with DS of maternal origin (49% DS-CHD^+^ mothers/51% DS-CHD^−^ mothers). LINE-1 DNA methylation was analyzed in peripheral blood lymphocytes by quantification of LINE-1 methylation using the MethyLight method. *MTHFR* C677T polymorphism genotyping was performed using PCR-RFLP.

**Results:** LINE-1 methylation was not significantly different between DS-CHD^+^ and DS-CHD^−^ mothers (*P* = 0.997). Combination of *MTHFR* C677T genotype/diet and BMI were significant independent predictors of LINE-1 DNA methylation in DS-CHD^+^ mothers (β −0.40, *P* = 0.01 and β −0.32, *P* = 0.03, respectively). In the analyzed multivariate model (model *P* = 0.028), these two factors explained around 72% of the variance in LINE-1 DNA methylation in mothers of children with DS and CHD. The group with the highest BMI (≥30 kg/m2) had significantly lower LINE-1 methylation than the group with normal BMI (Bonferroni *post hoc P* = 0.03) and the overweight group (Bonferroni *post hoc P* = 0.04). The lowest LINE-1 DNA methylation values were found in DS-CHD^+^ mothers with the CT+TT genotype and a low-folate diet; the values were significantly lower than the values in mothers with the CC genotype and a folate-rich diet (Bonferroni *post hoc P* = 0.04).

**Conclusion:** Association between maternal LINE-1 methylation and CHD in children with DS was not found. Study showed that the *MTHFR* genotype/diet combination and BMI were significantly associated with LINE-1 methylation in mothers of children with DS-CHD^+^. These results highlight the need for a multifactorial approach to assess the roles of endogenous and exogenous maternal factors in maternal LINE-1 DNA methylation and the consequent pathologies in children. More extensive studies in a larger sample may help elucidate these relationships.

## Introduction

Congenital heart defects (CHDs) are the most common birth defects in humans, with a prevalence of 0.8% (Dolk et al., 2011; Van Der Linde et al., 2011). The etiology of most CHDs is unknown but is thought to involve multiple genetic, epigenetic, environmental, and lifestyle factors (Botto et al., 2003; Pierpont et al., 2007; Dolk et al., 2011; Van Der Linde et al., 2011; Sun et al., 2015). Only about 15 to 20% of CHDs can be attributed to known causes, with 5 to 10% of cases with CHDs showing chromosomal abnormalities (Botto and Correa, 2003; Dolk et al., 2011). Trisomy 21 (OMIM 190685), which results in Down syndrome (DS), shows the highest association with major heart abnormalities, which are present in approximately 40 to 60% of individuals with DS. Such CHDs typically involve septal defects such as atrial septal defects, ventricular septal defects, and complete atrioventricular canal defects (Freeman et al., 2008; Marder et al., 2015). In addition to the direct effects of the chromosomal abnormality, maternal genotype, diet, and lifestyle factors, along with environmental exposures, may be involved in the development of heart anomalies in individuals with DS. Foremost among these maternal risk factors are folic acid deficiency and genetic variations of folate pathway genes, such as the methylenetetrahydrofolate reductase gene (*MTHFR*) (Brandalize et al., 2009; Hobbs et al., 2010; Coppedè, 2015; Asim et al., 2017). Altered maternal DNA methylation is suggested to be an underlying mechanism in the development of birth defects, including CHDs (Blom et al., 2006; Chowdhury et al., 2011; Barua and Junaid, 2015; Serra-Juhé et al., 2015; Spearman, 2017). Some risk factors have been proposed to modulate DNA methylation, including aging, body mass index (BMI), cigarette smoking, alcohol intake, folate deficiency, *MTHFR* polymorphisms, and hyperhomocysteinemia patterns (Chowdhury et al., 2011; Flom et al., 2011; Terry et al., 2011; Zacho et al., 2011; Delgado-Cruzata et al., 2015; Marques-Rocha et al., 2016; Mendelson et al., 2017; Wahl et al., 2017; Liu et al., 2018).

During the last decade, quantitative measurement of the methylation status of long interspersed nucleotide element-1 (LINE-1) in white blood cells (WBCs) has been used as a surrogate measure of global DNA methylation and as a potential biomarker in a variety of diseases (Weisenberger et al., 2005; Chowdhury et al., 2011). Maternal LINE-1 hypomethylation has been linked to an increased risk of non-syndromic CHD, particularly septal defects (Chowdhury et al., 2011). We previously found significantly lower levels of LINE-1 methylation in the mothers of children with DS than in the mothers of healthy children (Božović et al., 2015). However, the relationship between LINE-1 methylation and DS-associated CHD has not yet been investigated.

Thus, the aim of the present study was to assess (i) the association between maternal LINE-1 methylation and the occurrence of CHD in children with DS and (ii) the association of endogenous maternal factors (*MTHFR* C677T polymorphism and maternal age), and exogenous maternal factors (cigarette smoking, alcohol intake, medication use, body mass index, and dietary habits, such as folate intake) with LINE-1 methylation in mothers of children with DS and CHD.

## Materials and Methods

### Study Participants

The study population consisted of 90 mothers of children with maternally derived full trisomy 21. All participants were the same ethnicity (Caucasian); 49% (44/90) had children with DS and CHD (DS-CHD^+^ mothers), and 51% (46/90) had children with DS without CHD (DS-CHD^−^ mothers). There was a septal defect in 82% (36/44) of the children with DS-CHD^+^, a patent foramen ovale in 11% (5/44), patent ductus arteriosus in 5% (2/44), and persistent truncus arteriosus in 2% (1/44). Information about CHD was obtained from each child’s medical records. Maternal blood samples were collected in collaboration with DS associations in larger cities in Croatia (Rijeka, Zagreb, Pula, Zadar, Split, Karlovac, Čakovec, and Osijek). The Ethics Committee of the School of Medicine, University of Rijeka, reviewed and approved all study protocols (reference number: 2170-24-01-13-04). Written informed consent was provided by all participants prior to participation in the study in accordance with the Declaration of Helsinki. Before the sampling, the mothers were asked to complete a specially created questionnaire that asked about demographic data, weight and height, intake of folate-rich foods, cigarette smoking, alcohol intake, and medication use. The questionnaire was adapted from a food frequency questionnaire that has been validated for Croatian women (Colić et al., 2009).

### Genetic Analysis

Genomic DNA was extracted from peripheral blood leukocytes using the QIAamp DNA Blood FlexiGene DNA Kit (Qiagen, Hilden, Germany). Quantification of genomic DNA was performed using a spectrophotometer (BioMateTM3, Thermo Electron Corporation, United States). The parental origin of trisomy 21 was determined as described previously (Vraneković et al., 2012). The *MTHFR* C677T polymorphism was evaluated using polymerase chain reaction–restriction fragment length polymorphism (PCR-RFLP) (Coppedè et al., 2006). Using the EpiTect Bisulfite Kit (Qiagen, Hilden, Germany), 500 ng of genomic DNA was subjected to sodium bisulfite modification and resuspended in 30 μl of TE buffer. Bisulfite-treated DNA was diluted 10×, and 8.18 μl was used for each real-time PCR reaction. All samples were stored at −20°C until analysis.

LINE-1 DNA methylation was analyzed by quantifying LINE-1 methylation using previously developed and validated MethyLight methodology, which precision and reproducibility have been well described (Eads et al., 2000; Weisenberger et al., 2005; Chowdhury et al., 2011; Božović et al., 2015). The PCR primers and probes for LINE-1 and for Alu sequences (ALU-C4) (Applied Biosystems, Forest City, CA, United States) were designed/validated (Weisenberger et al., 2005) and described previously (Weisenberger et al., 2005; Božović et al., 2015). The LINE-1 primers lack CpG sites and the specific TaqMan MGB probes (corresponding to the methylated and unmethylated LINE-1 sequence after bisulphite treatment) were used in real-time PCR (Applied Biosystems, Forest City, CA, United States). An Alu-based real-time PCR control reaction was performed in parallel with each LINE-1 real-time PCR reaction to normalize DNA input, as previously described (Weisenberger et al., 2005; Božović et al., 2015). PCR reactions were performed with a final reaction volume of 25 μl in sealed 96-well plates on the ABI 7500 Real-Time PCR System (Applied Biosystems). The PCR reactions and cycle conditions for the LINE-1 and ALU-C4 assays were described previously (Božović et al., 2015). Real-time PCR was performed in duplicate for each sample. The standard curve was established using EpiTect Methylated Control DNA and EpiTect Unmethylated Control DNA (Qiagen), as described previously (Božović et al., 2015). After PCR amplification, the data were read using SDS 1.4.0 software (Applied Biosystems). The percentage of methylated reference (PMR) and the percentage of unmethylated reference (PUR) were calculated, and the final percentage of LINE-1 DNA methylation was calculated according to the formula PMR/(PMR+PUR) × 100 (Eads et al., 2000; Weisenberger et al., 2005; Božović et al., 2015). After all runs we randomly repeated runs for 10% of all samples and we have complete reproducibility of the results.

### The Influence of Endogenous and Exogenous Factors on Maternal LINE1 DNA Methylation

The analysis included maternal age and BMI as continuous variables and folate supplement intake, cigarette smoking, alcohol intake, and medication use as categorical variables. BMI was also studied as a categorical variable (“WHO BMI”), and four groups were defined according to WHO classification: underweight, normal, overweight, and obese. Because the impact of folic acid intake is modified by genes that code for enzymes involved in folate metabolism, the analysis also included the variable “*MTHFR* C677T genotype/diet,” which was used to indicate the combination of the *MTHFR* C677T polymorphism and dietary folate intake (rich or poor) (Friso et al., 2002; Castro et al., 2004). A folate-rich diet was defined as the consumption of at least three folate-rich foods (green leafy vegetables, legumes, veal liver, fruit, corn flakes, muesli) at least 2–3 times each week; lower intake was considered a low-folate diet. Mothers who consumed folic acid daily from 4 weeks before conception until 8 weeks after conception were considered to be periconceptional folic acid users. The questionnaire asked about cigarette smoking, alcohol intake, and medication use both (i) during the first 6 weeks of pregnancy and (ii) currently. Mothers who consumed one glass of wine or one glass of beer or one small strong alcoholic drink at least one time per week were defined as alcohol consumers. Those who smoked cigarettes daily or occasionally were classified as smokers.

### Statistical Analysis

Differences in the frequencies between the investigated groups of participants were estimated by the chi-square test. Continuous variables with skewed distribution between groups were compared using the Mann–Whitney *U*-test. Normally distributed continuous variables were compared using analysis of variance (ANOVA) with the Bonferroni *post hoc* test and are reported as means and standard deviations (SDs). Logistic regression was used to determine the odds ratio and 95% confidence interval for the association between *MTHFR* C677T polymorphism and the occurrence of CHD in children with DS. Multivariate regression analysis was used to estimate the effects of the endogenous and exogenous factors included in the model on LINE1 DNA methylation (*MTHFR* C677T polymorphism, age, BMI, dietary intake of folate, intake of folate supplements, smoking, alcohol intake, and medication use), and the results are presented as regression coefficient (beta) values plus the standard error (SE). Statistical significance was considered for *P <* 0.05. Data analysis was performed using Statistica for Windows 10.0 (StatSoft, Tulsa, OK, United States).

## Results

Table 1 shows the characteristics of the study participants. DS-CHD^+^ mothers were significantly younger than DS-CHD^−^ mothers (*P* = 0.003). The frequency of mothers who smoked cigarettes during the first 6 weeks of pregnancy was significantly higher in DS-CHD^+^ mothers than in DS-CHD^−^ mothers (*P* = 0.005).

**Table 1:** Characteristics of mothers of children with Down syndrome (DS) with or without congenital heart defects (CHDs).

	DS-CHD^+^ mothers	DS-CHD^−^ mothers	*P*-value
Median age, years (min–max)	37 (24–59)	42 (28–64)	0.00
Median BMI (min–max)	24.19 (17.19–35.43)	26.01 (17.71–40.40)	0.09
**Diet N (%)**			
Folate-rich	16 (36)	21 (46)	0.24
Low-folate	28 (64)	45 (54)	
**Intake of folic acid supplements N (%)**			
No	25 (57)	33 (72)	0.14
Yes	19 (43)	13 (28)	
**Periconceptional folic acid intake**			
No	41 (93)	46 (100)	0.17
Yes	3 (7)	0 (0)	
**Smoking during the first 6 weeks of pregnancy N (%)**			
No	15 (34)	29 (63)	0.00
Yes	29 (66)	17 (37)	
**Currently smoking N (%)**			
No	27 (71)	33 (72)	0.21
Yes	17 (39)	13 (28)	
**Alcohol intake during the first 6 weeks of pregnancy N (%)**			
No	31 (70)	33 (72)	0.54
Yes	13 (30)	13 (28)	
**Current alcohol intake N (%)**			
No	32 (73)	37 (80)	0.27
Yes	12 (30)	9 (20)	
**Medication use during the first 6 weeks of pregnancy N (%)**			
No	38 (86)	39 (85)	0.53
Yes	6 (14)	7 (15)	
**Current medication use N (%)**			
No	42 (95)	43 (93)	0.52
Yes	2 (5)	3 (7)	

*P-values were determined using the Mann–Whitney test or the chi-square test.*

Supplementary Table S1 shows the allele and genotype frequencies of the *MTHFR* C677T polymorphism in DS-CHD^+^ and DS-CHD^−^ mothers. There were no significant differences between the two groups.

### Maternal LINE1 DNA Methylation

There was no significant difference in LINE-1 methylation between DS-CHD^+^ mothers (median: 95.30%; min–max: 88.68–99.90%) and DS-CHD^−^ mothers (median: 95.51%; min–max: 79.13–99.73%) (*P* = 0.997). The difference in LINE-1 methylation between these groups remained non-significant after adjusting for two factors that were significantly different between these groups, namely maternal age and smoking during the first 6 weeks of pregnancy (adjusted OR = 1.03, 95% CI: 0.876–1.173, *P* = 0.853).

We have shown in Table 2 the values of LINE-1 methylation in DS-CHD^+^ mothers according to WHO BMI categories. Those in the highest WHO BMI category (≥30 kg/m2) had significantly lower LINE-1 methylation than those in either the normal WHO BMI category (Bonferroni *post hoc P* = 0.03) or the overweight WHO BMI category (Bonferroni *post hoc P* = 0.04). Table 3 lists the values for LINE-1 methylation in DS-CHD^+^ mothers according to *MTHFR* C677T genotype/diet combinations. The lowest LINE-1 DNA methylation values were observed in mothers with the CT+TT genotype and a low-folate diet. We performed multivariate regression analysis in order to evaluate the independent effect of the investigated parameters on LINE-1 DNA methylation in DS-CHD^+^ mothers. Among the investigated predictors (Table 4), only the *MTHFR* C677T genotype/diet combination and BMI were significantly independently associated with LINE-1 DNA methylation in DS-CHD^+^ mothers (β -0.40, *P* = 0.01 and β-0.32, *P* = 0.03, respectively).

**Table 2:** LINE-1 methylation in mothers of children with DS with congenital heart defects (CHDs) according to World Health Organization (WHO) body mass index (BMI) category.

WHO-BMI	DNA LINE1 methylation (%) Means	DNA LINE1 methylation, SE	*N*
18.5	93.77	1.87	2
18.5–24.9	95.86	0.55	23
25–29.9	96.08	0.76	12
≥30	92.50^∗^	1.00	7

*ANOVA P = 0.02; ^∗^Bonferroni post hoc, BMI ≥ 30 vs. BMI 25–29.9, P = 0.04; BMI ≥ 30 vs. BMI 18.5–24.9, P = 0.03, N = 44.*

**Table 3:** LINE-1 methylation in mothers of children with DS with congenital heart defects (CHDs) according to the *MTHFR* C677T genotype/diet combination.

*MTHFR* C677T genotype/diet	DNA LINE1 methylation (%) Means	DNA LINE1 methylation SE	*N*
CC/folate-rich diet	97.69	0.89	9
CT+TT/folate-rich diet	94.94	1.01	7
CC/folate-poor diet	94.68	0.77	12
CT+TT/folate-poor diet	94.55^∗^	0.67	16

*ANOVA P = 0.04, ^∗^Bonferroni post hoc CT+TT/folate-poor diet, N = 44.*

**Table 4:** Multivariate analysis of predictors that influence LINE-1 methylation in mothers of children with DS with congenital heart defects (CHDs).

Predictors included in the analysis	Beta	Standard error of beta	*P*-level
Currently smoking	0.10	0.16	0.53
Current alcohol intake	0.08	0.15	0.57
Current medication use	−0.20	0.14	0.17
BMI	−0.32	0.14	0.03
Periconceptional folic acid intake	−0.01	0.15	0.96
*MTHFR* C677T genotype/diet	−0.40	0.15	0.01
Age of mother	−0.22	0.15	0.14

*R = 0.58, R^2^ = 0.34, adjusted R^2^ = 0.21, P < 0.028.*

In addition, we determined the LINE-1 methylation values in mothers of children with DS and septal defects. We found that the *MTHFR* C677T genotype/diet combination significantly influenced LINE-1 DNA methylation (Table 5). There were no statistically significant associations with LINE-1 methylation identified in DS-CHD^−^ mothers (Supplementary Tables S2–S4).

**Table 5:** Multivariate analysis of predictors that influence LINE-1 methylation in mothers of children with DS with septal defects.

Predictors included in the analysis	Beta	Standard error of beta	*P*-value
Intercept			0.00
BMI	−0.27	0.15	0.08
*MTHFR* C677T genotype/diet	−0.36	0.15	0.02

*R = 0.47, R^2^ = 0.23, adjusted R^2^ = 0.18, P < 0.015.*

## Discussion

To the best of our knowledge, this is the first study to investigate the impact of endogenous maternal factors (*MTHFR* C677T polymorphism and maternal age) and exogenous maternal factors (cigarette smoking, alcohol intake, medication use, body mass index, dietary habits such as folate intake) on LINE-1 methylation in the mothers of children with DS regarding the presence of DS-associated CHDs, particularly regarding the presence of septal defect. The molecular mechanisms that underlie the epigenetic regulation of gene transcription are independent of DNA sequence, but they do depend on environmental stimuli, such as periconceptional maternal supplementation, diet, and the in utero environment (Barua and Junaid, 2015; Toriyama et al., 2017). The morphological processes that accompany embryonic heart development remain largely unknown, but multiple genetic, epigenetic, environmental, and lifestyle factors likely influence this process (Pierpont et al., 2007; Van Der Linde et al., 2011; Eriksson, 2016; Grunert et al., 2016; Toriyama et al., 2017).

We found that BMI and the *MTHFR* genotype/diet combination were significantly associated with variations in LINE-1 DNA methylation in DS-CHD^+^ mothers. Lower LINE1 DNA methylation values were significantly associated with the genotype containing the *MTHFR* 677T allele in combination with a low folate diet as well as with higher BMI, which is in accordance with previous studies (Friso et al., 2002; Castro et al., 2004; Piyathilake et al., 2011; Cai et al., 2014). Notably, it was reported previously that higher BMI is associated with lower LINE-1 methylation values in women of childbearing age (Piyathilake et al., 2011). The association between a maternal BMI that is higher than recommended by WHO and the occurrence of CHD in their offspring is well documented (Stothard et al., 2009; Piyathilake et al., 2011; Block et al., 2013; Cai et al., 2014). It is in line with our finding of significant influence of BMI only in the DS-CHD^+^ mothers. The mechanism by which BMI influences the development of CHD is not well understood, but it is thought that obesity is linked to lower concentrations of folate in the blood as well as with undiagnosed diabetes, both of which are maternal risk factors for CHD development (Hötzel, 1986; Becerra et al., 1990; Towner et al., 1995; Casanueva et al., 2000; Stothard et al., 2009; Hobbs et al., 2010). Likewise, increasing evidence suggests that folate metabolism and the resulting epigenetic modifications may contribute to the occurrence of CHD in individuals with DS (Brandalize et al., 2009; Elsayed et al., 2014; Coppedè, 2015).

DNA synthesis and methylation, processes that are folate-dependent, increase during pregnancy (Oommen et al., 2005). Folate pathway genes have been extensively investigated in regard to their association with CHD (Van Beynum et al., 2006, 2007; Wang et al., 2013; Elsayed et al., 2014). The MTHFR enzyme plays a key role in the regulation of folate availability in DNA synthesis and methylation. The C677T is one of the most important functional polymorphisms of the MTHFR gene (Frosst et al., 1995). Numerous studies have investigated the association between the *MTHFR* C677T polymorphism and the risk of CHDs, but the results have been inconsistent (Hobbs et al., 2005; Zhu et al., 2006; Brandalize et al., 2009; Božović et al., 2011). Meta-analyses showed that maternal *MTHFR* C677T polymorphism may contribute to the risk of CHDs (Wang et al., 2013; Xuan et al., 2014; Yang et al., 2018). Research on the relationship between the maternal *MTHFR* genotype and the development of CHD in children with DS has also yielded conflicting results (Brandalize et al., 2009; Hobbs et al., 2010; Božović et al., 2011; Elsayed et al., 2014; Coppedè, 2015). There has not yet been a meta-analysis, but almost all studies have shown that the *MTHFR* C677T genotype may be a maternal risk factor for CHD in children with DS, particularly if the mothers did not consume folic acid during the periconceptional period (Brandalize et al., 2009; Elsayed et al., 2014).

It is well established that increased folate intake can neutralize the impact of the *MTHFR* C677T polymorphism and restore normal enzyme activity (Guenther et al., 1999; Kluijtmans et al., 2003). Thus, a number of studies suggest that periconceptional maternal folic acid use has a protective effect on the occurrence of CHD in offspring, particularly for septal defects (Czeizel et al., 2001; Botto et al., 2003; Van Beynum et al., 2010). In our study, the periconceptional use of folate was not significantly associated with the level of LINE-1 methylation by itself. However, dietary folate intake in combination with the *MTHFR* C677T genotype showed a significant association with LINE-1 methylation levels. Moreover, our results revealed that the values of LINE-1 DNA methylation in DS-CHD^+^ mothers, as well as in the mothers of children with DS and septal defects, were clearly stratified according to the *MTHFR* C677T genotype/diet combination: the lowest values were observed in mothers with the CT+TT genotype and a low-folate diet, and the highest levels were observed in mothers with the CC genotype and a folate-rich diet. Chowdhury et al. also reported significant LINE-1 hypomethylation in women with children affected by septal defects (Chowdhury et al., 2011).

We found that the *MTHFR* C677T genotype/low folate diet combination was significantly associated with LINE-1 hypomethylation in mothers with children with DS who developed septal defects, and our previous study showed that significant LINE-1 hypomethylation (compared to controls) in the mothers of children with DS was itself significantly associated with the *MTHFR* C677T genotype/diet combination (Božović et al., 2015). Since the maternal environment potentially affects the fetus during pregnancy (Dimasuay et al., 2016), it is important that the analysis include as many factors as possible that could influence fetal development. In the present study, the *MTHFR* C677T genotype/diet combination and BMI showed a univariate association with LINE-1 DNA methylation in DS-CHD^+^ mothers. In addition, these parameters were independently associated with of LINE-1 DNA methylation in a multivariate analysis that included other maternal risk factors, such as smoking, periconceptional folic acid intake, medication and alcohol use, and age. In the multivariate model (model *P <* 0.028), these two factors explained around 72% of the variance in LINE-1 DNA methylation in DS-CHD^+^ mothers. Factors like cigarette smoking, alcohol intake, and age showed no associations with LINE-1 methylation status, which is in accordance with other studies (Terry et al., 2011; Zhang et al., 2011; Jones et al., 2015). Medication use was not significantly associated with LINE-1 DNA methylation, although only 6% of the participants were taking medications, and these belonged to several different therapeutic groups. Thus, we do not currently have enough data to discuss whether certain medications influence LINE-1 DNA methylation.

This study has several limitations. Notably, approximately 1/3 of fetuses with trisomy 21 are lost during early pregnancy (Savva et al., 2006). Thus, the true prenatal incidence of CHD in fetuses with trisomy 21 is unknown, and we can only speculate that those lost during early pregnancy may be more affected by CHDs than those who are born alive. Several studies have indicated that the increase of the prevalence of cardiac anomalies with decreasing fetal gestational age contributes to higher numbers of miscarriages (Gerlis, 1985; Tomek et al., 2009). Tomek et al. reported that the spectrum of CHDs that are diagnosed prenatally differs significantly from the spectrum of CHDs diagnosed postnatally in that there is a markedly higher proportion of additional abnormalities associated with those who are diagnosed prenatally (Tomek et al., 2009). Thus, we would have a clearer picture of the impact of maternal LINE-1 DNA methylation on DS-associated CHDs if we could analyze the maternal LINE-1 DNA methylation values during pregnancy (and thus during organogenesis) for all conceived fetuses with trisomy 21, since during this time, altered maternal DNA methylation would exert the greatest effects. It is possible that increases in cellular proliferation and carbon metabolism during pregnancy, as well as an increased demand for methyl groups during embryonic development, could have an even greater influence on maternal LINE-1 DNA methylation and contribute to the development of CHD in offspring. Given the rare prevalence of DS-associated CHD, the resources and sample size required to conduct such a study, and the difficulty in enrolling women before conception and monitoring them until the completion of their pregnancy, it would be very challenging to conduct this type of study. Also, analysis of other class of repetitive elements, such as ALU, might give additional information on the global DNA methylation in mothers of children with DS with regard to presence of CHD. It was suggested that Alu might be more informative in background where the LINE-1 hypomethylation might be influenced by genomic instability, carcinogenesis or aging (Erichsen et al., 2018).

In conclusion, we have not found the association of maternal LINE-1 methylation with CHD in children with DS. Yet, we have found significant association of the *MTHFR* genotype/diet combination and BMI with LINE-1 methylation in mothers of children with DS-CHD and in mothers of children with DS and septal defects. These results strongly support the need for a multifactorial approach in analysis of endogenous and exogenous maternal factors that could affect or are associated with maternal LINE-1 DNA methylation and the consequent pathologies in children. More extensive studies in a larger sample may help to validate these results while functional studies are inevitable to elucidate the causality and mechanisms of action of proposed factors.

## Datasets Are Available on Request

The raw data supporting the conclusions of this manuscript will be made available by the authors, without undue reservation, to any qualified researcher.

## Author Contributions

IBB, AS, MŽ, and JV processed the experimental data, performed the analysis, and drafted the manuscript. BB-M, AS, and MŽ were involved in planning and supervised the work. VM-S aided in collecting the patients. All authors discussed the results and commented on the manuscript.

## Conflict of Interest Statement

The authors declare that the research was conducted in the absence of any commercial or financial relationships that could be construed as a potential conflict of interest.

## Abbreviations

BMI: body mass index
CHD: congenital heart defects
DS: Down syndrome
LINE-1: long interspersed nucleotide element-1
MTHFR: methylenetetrahydrofolate reductase
PCR: polymerase chain reaction
PMR: percent of methylated reference
PUR: percent of unmethylated reference
WBC: white blood cells.
