# Supplementary material for: Maternal LINE-1 DNA Methylation and Congenital Heart Defects in Down Syndrome
Source: Front Genet. 2019 Feb 6;10:41. doi: 10.3389/fgene.2019.00041 (PMC6372553; doi:10.3389/fgene.2019.00041)
Supplement: Supplementary file 1 [file Data_Sheet_1.docx]

Supplementary tables

Table S1. Allele and genotype frequencies of the *MTHFR* C677T polymorphism in mothers of children with Down syndrome (DS) with or without congenital heart defects (CHDs)

| *MTHFR* C677T | DS-CHD^+^ mothers  N (%) | DS-CHD^-^ mothers  N (%) | *P*-value  OR (95%CI) |
| --- | --- | --- | --- |
| Genotype |  | | |
| CC | 21 (48) | 22 (48) | Reference |
| CT | 19 (43) | 18 (39) | 0.82  1.11 (0.46–2.66) |
| TT | 4 (9) | 6 (13) | 0.62  0.70 (0.17–2.83) |
| CT+TT | 23 (52) | 24 (52) | 1.00  0.44 (0.44–2.30) |
| Allele |  | | |
| C | 61 (69) | 62 (67) | Reference |
| T | 27 (31) | 30 (33) | 0.78  0.91 (0.49–1.71) |

OR, odds ratio; 95%CI, 95% confidence interval; *P*-values were determined using the chi-square test.

Table S2. LINE-1 methylation in mothers of children with Down syndrome (DS) without congenital heart defects (CHDs) according to World Health Organization (WHO) body mass index (BMI) category

| WHO-BMI | DNA  LINE1 methylation (%)  Means | DNA  LINE1 methylation,  standard error | N |
| --- | --- | --- | --- |
| <18.5 | 94.53 | 2.15 | 3 |
| 18.5–24.9 | 94.41 | 0.90 | 17 |
| 25–29.9 | 95.60 | 0.90 | 17 |
| ≥30 | 94.96 | 1.24 | 9 |

ANOVA *P=*0.82; N=46

Table S3. LINE-1 methylation in mothers of children with Down syndrome (DS) without congenital heart defects (CHDs) according to the *MTHFR* C677T genotype/diet combination

| *MTHFR* C677T genotype/diet | DNA  LINE1 methylation (%)  Means | DNA  LINE1 methylation  standard error | N |
| --- | --- | --- | --- |
| CC/folate-rich diet | 95.18 | 1.07 | 12 |
| CT+TT/folate-rich diet | 95.17 | 1.23 | 9 |
| CC/folate-poor diet | 95.93 | 1.17 | 10 |
| CT+TT/folate-poor diet | 94.03 | 0.95 | 15 |

ANOVA *P=*0.64, N=46

Table S4. Multivariate analysis of predictors that influence LINE-1 methylation in mothers of children with Down syndrome (DS) without congenital heart defects (CHDs)

| Predictors included in the analysis | Beta | Standard error of beta | *P*-level |
| --- | --- | --- | --- |
| Currently smoking | -0.09 | 0.17 | 0.58 |
| Current alcohol intake | -0.08 | 0.16 | 0.62 |
| Current medication use | -0.01 | 0.17 | 0.91 |
| BMI | 0.177 | 0.18 | 0.34 |
| Periconceptional folic acid intake | 0.20 | 0.19 | 0.29 |
| *MTHFR* C677T genotype/diet | 0.06 | 0.16 | 0.72 |
| Age of mother | -0.04 | 0.21 | 0.84 |

R=0.31, R²=0.10, adjusted R²=NA, *P=*0.78
